# Risk of Suicide Related to Mental Disorders in Catalonia, Spain: A Population Registry-Based Cohort Study

**DOI:** 10.1192/j.eurpsy.2025.334

**Published:** 2025-08-26

**Authors:** P. Mortier, L. Latorre-Moreno, F. Amigo, M. López, A. Portillo-Van Diest, L. Ballester, J. Alonso, G. Vilagut

**Affiliations:** 1 Health Services Research Group, Hospital del Mar Research Institute, Barcelona; 2 Centro de Investigación Biomédica en Red de Epidemiología y Salud Pública, Instituto de Salud Carlos III (CIBERESP, ISCIII), Madrid, Spain; 3 Department of Population Health Sciences, Duke University School of Medicine, Durham, United States; 4 Department of Experimental and Health Sciences, Pompeu Fabra University, Barcelona, Spain

## Abstract

**Introduction:**

Registry-based studies are efficient to investigate population mental health and suicide risk, but are largely absent in Europe outside of Scandinavia and the UK.

**Objectives:**

To investigate suicide risk associated with mental disorders in the Catalan population (7.6 million), stratified by gender and history of psychiatric hospitalization.

**Methods:**

Population-representative retrospective registry-based cohort study including 764,938 Catalan residents in the period 2014-2019. Data sources included suicide mortality, electronic health registries from five healthcare settings, and administrative data. Suicide deaths were identified through judicial death registers using ICD-10 codes X60-X84. ICD-9CM and ICD10CM codes from all inpatient and outpatient healthcare contacts were used to categorize 109 mental disorders. Age-sex standardized mortality ratios (SMRs) were calculated using indirect standardization, with expected deaths based on official general population mortality rates in Catalonia.

**Results:**

Suicide risk was significantly elevated among Catalan residents diagnosed with any mental disorder (SMR [95%CI] = 1.6 [1.3-1.9] for females; SMR = 1.8 [1.6-2.0] for males). In females, suicide risk was highest for sedative or hypnotic abuse (SMR = 46.1 [3.7-88.5]), cocaine abuse (SMR = 42.8 [9.0-76.6]), borderline personality disorder (SMR = 33.0 [10.7-55.3]), polysubstance abuse (SMR = 32.9 [2.1-63.8]), and mental disorder not otherwise specified (SMR = 24.9 [11.7-38.1]). In males, risk was highest for obsessive-compulsive disorder (SMR = 20.2 [10.7-29.8]), acute and transient psychotic disorders (SMR = 17.9 [1.1-34.8]), mental disorder not otherwise specified (SMR = 17.2 [10.1-24.3]), paranoid schizophrenia (SMR = 16.8 [9.4-24.1]), and opioid abuse (SMR = 16.1 [1.6-30.6]). Suicide risk was substantially elevated in individuals with a history of psychiatric hospitalization (SMR = 18.3 [15.5-21.2]] for females; SMR = 13.4 [12.0-14.8] for males). In females with psychiatric hospitalization history, risk was highest for dependence on stimulants other than cocaine (SMR = 105.4 [11.6-199.3]), attention deficit hyperactivity disorder (SMR = 86.2 [19.2-153.2]), polysubstance abuse (SMR = 66.0 [33.6-98.3]), opioid abuse (SMR = 60.1 [2.1-118.1]), and cocaine abuse (SMR = 57.2 [32.6-81.8]). In males with psychiatric hospitalization history, risk was highest for obsessive-compulsive disorder (SMR = 45.7 [30.6-60.8]), schizoid personality disorder (SMR = 36.9 [13.5-60.2]), unspecified disorders of adult personality and behaviour (SMR = 35.0 [9.6-60.3]), schizotypal disorder (SMR = 34.3 [6.7-61.9]), and histrionic personality disorder (SMR = 32.5 [1.2-63.9]).

**Conclusions:**

Risk of suicide in the Catalan population varies substantially by mental disorder type, gender, and psychiatric hospitalization history, highlighting the need for targeted and diversified prevention strategies.

**Disclosure of Interest:**

P. Mortier Grant / Research support from: Miguel Servet CP21/00078 (ISCIII co-funded by the ESF+); project 202220-30-31 (Fundació la Marató de TV3); PI22/00107 (ISCIII, cofunded by the European Union); AC22/00006 (ISCIII and the European Union NextGenerationEU, Mecanismo Para la Recuperación y la Resiliencia), L. Latorre-Moreno: None Declared, F. Amigo: None Declared, M. López: None Declared, A. Portillo-Van Diest Grant / Research support from: PFIS FI23/00004 (ISCIII co-funded by the ESF+), L. Ballester: None Declared, J. Alonso Grant / Research support from: AGAUR 2021 SGR 00624; CB06/02/0046 (CIBERESP - ISCIII), G. Vilagut: None Declared

